# Sleep Apnea-Hypopnea Quantification by Cardiovascular Data Analysis

**DOI:** 10.1371/journal.pone.0107581

**Published:** 2014-09-15

**Authors:** Sabrina Camargo, Maik Riedl, Celia Anteneodo, Jürgen Kurths, Thomas Penzel, Niels Wessel

**Affiliations:** 1 Department of Physics, Humboldt-Universität zu Berlin, Berlin, Germany; 2 EMAp, Fundação Getúlio Vargas, Rio de Janeiro, Brazil; 3 Department of Physics, PUC-Rio, Rio de Janeiro, Brazil; 4 National Institute of Science and Technology for Complex Systems, Rio de Janeiro, Brazil; 5 Potsdam Institute for Climate Impact Research, Potsdam, Germany; 6 Institute for Complex Systems and Mathematical Biology, University of Aberdeen, Aberdeen, United Kingdom; 7 Sleep Center, Charité University Hospital, Berlin, Germany; University of Adelaide, Australia

## Abstract

Sleep disorders are a major risk factor for cardiovascular diseases. Sleep apnea is the most common sleep disturbance and its detection relies on a polysomnography, i.e., a combination of several medical examinations performed during a monitored sleep night. In order to detect occurrences of sleep apnea without the need of combined recordings, we focus our efforts on extracting a quantifier related to the events of sleep apnea from a cardiovascular time series, namely systolic blood pressure (SBP). Physiologic time series are generally highly nonstationary and entrap the application of conventional tools that require a stationary condition. In our study, data nonstationarities are uncovered by a segmentation procedure which splits the signal into stationary patches, providing local quantities such as mean and variance of the SBP signal in each stationary patch, as well as its duration 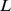. We analysed the data of 26 apneic diagnosed individuals, divided into hypertensive and normotensive groups, and compared the results with those of a control group. From the segmentation procedure, we identified that the average duration 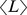, as well as the average variance 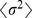, are correlated to the apnea-hypoapnea index (AHI), previously obtained by polysomnographic exams. Moreover, our results unveil an oscillatory pattern in apneic subjects, whose amplitude 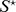 is also correlated with AHI. All these quantities allow to separate apneic individuals, with an accuracy of at least 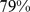. Therefore, they provide alternative criteria to detect sleep apnea based on a single time series, the systolic blood pressure.

## Introduction

Sleep disturbances (e.g., sleep apnea, insomnia, restless legs syndrome, sleep walking, sleep terror) deserve serious attention since they constitute an important risk factor for cardiovascular disorders such as hypertension, cardiac ischemia, sudden cardiac death, and stroke [1]–[3]. Blood pressure, heart rate variability, respiratory variability, and other cardiorespiratory data could be useful to detect sleep disturbances, especially the most common sleep apnea. Individuals who suffer from this kind of disorder usually present daytime sleepiness, loud snoring and restless sleep. A sleep apnea event is defined as a break in the airflow that lasts at least 10 secs. If the air flow is less than 50% of normal, the resulting airflow limitation is called hypopnea [4]. When there is no inspiratory effort, then the event is classified as central. If respiratory effort is made against an upper airway obstruction, then the apnea event is classified as obstructive. Sleep apnea events can also be of a mixed type.

In order to obtain a sleep profile, the common practice is to combine records collected by means of different exams: electroencephalography (EEG), electromyography (EMG), and electro-oculography (EOG). This set of tests produces a polysomnography, from which a scoring of sleep stages is visually evaluated, assigning to each stage the pattern found in consecutive 30-second-long epochs of the EEG, EMG, and EOG recordings. The resulting succession of discrete sleep stages is referred to as a hypnogram and supports diagnostic decisions [5]. Signals of airflow respiratory effort such as abdominal movement and oxygen saturation of the blood are also used in diagnosis of sleep apnea [6], which, as mentioned before, requires combined records. Therefore, it would be desirable to evaluate sleeping through an alternative procedure consisting of simpler data recordings. This is the goal we pursue in the present work.

It is important to emphasize that cardiorespiratory time series are highly nonstationary, which restricts the use of standard tools of time series analysis. In this regard, Penzel et al. showed that changes in heart rate variability in obstructive apneas were better quantified by scaling analysis (using detrended fluctuation analysis) than by spectral methods [7], [8]. This is because, techniques such as fast Fourier transformation require stationarity in order to give a meaningful estimation of the spectral components of a time series [9]. Hence, we apply a nonparametric segmentation procedure to yield patches where stationarity is verified. Within each of these locally stationary data segments, the statistical moments of the signal, such as mean and variance, remain constant. Segmentation also provides the intrinsic time scales, through the duration of segment lengths. Moreover, by finding the stationary regimes, one might be able to identify changes in a time series, as those coming from the apnea occurrence.

## Materials and Methods

The study and the consent procedure for this research were approved by the ethics committee, Charité - Universitätsmedizin, in Berlin. Participants provided their written informed consent to participate in this study and the informed consent of all subjects was recorded in paper form. We analyzed data from 26 patients suffering from apnea-hypopnea, that is with apnea-hypopnea index (AHI, the average number of apnea events per hour) larger than 15, including obstructive, central, and mixed sleep apnea events. Most of the events are of the obstructive type, with only 4 subjects presenting a majority of central type events. The patients are then divided into two groups, according to their diurnal systolic blood pressure levels: 10 hypertensive subjects (HT) and 16 normotensive patients (NT). The mean values of the systolic blood pressure were 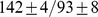 mmHg in HT and 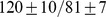 mmHg in NT. Hypertensive subjects were chosen for comparison, due to the known association between sleep disordered breathing events and autonomic reactions such as blood pressure increase [10]. Moreover, we considered a control group (C) with 7 non-apneic subjects. All three groups are age and sex matched, being all males with mean age of 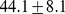 years (HT), 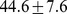 (NT), and 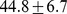 (C). Excluding criteria to select the 26 patients were heart rhythm disturbances, and other comorbid illnesses like diabetes. The apnea-hypopnea indexes in the groups are 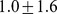 (C), 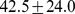 (NT) and 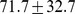 (HT). Regarding the measurement system, the polysomnographic system Embla N7000 was used. The ECG was recorded at 2 kHz. R peak extraction was done automatically with an accuracy of 0.5 ms.

The beat-to-beat-intervals from the ECG as well as the systolic blood pressure intervals were analyzed separately and filtered adaptively to exclude misclassifications and artifacts, e.g., premature beats [11]. The intervals between successive heartbeats (beat-to-beat intervals) were extracted from the electrocardiogram records [12]. All measurements were monitored during one night sleep. Blood pressure was "continuously" monitored (at a sampling rate of 200 Hz) with a finger cuff sensor (Portapres Model2, BMI-TNO). From the continuous blood pressure signal, the maximum value in each beat-to-beat interval was extracted, producing the time series of systolic blood pressure (SBP) on a beat-to-beat basis. Analogous procedure was followed by using minimum blood pressure values to extract the beat-to-beat diastolic blood pressure (DBP) [13]–[15]. Beat-to-beat intervals from blood pressure (BBI-BP) records and from electrocardiograms (BBI-EKG) were also analyzed. We observed similar results for SBP and DBP series, but SBP presents slightly better evaluation. We will concentrate on SBP in the further description.

We first dealt with the nonstationarity of the series performing a segmentation of the signal into stationary-like patches. The segmentation procedure used, which is based on the Kolmogorov-Smirnov (KS) statistics, is explained in Ref. [16]. Succinctly, this KS-segmentation is done through the following steps: given the time series, all points of the signal are considered as a potential cutting point, and we compute the Kolmogorov-Smirnov distance 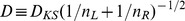, between the cumulative distributions of the points belonging to the two segments placed at the left and the right sides of the cutting point, with lengths 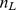 and 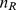, respectively. Thus, there will be one value of 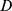 corresponding to a hypothetical cut at each point of the signal, and we determine the position 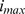 where 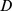 is maximal. Once we know the position 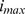 of the maximal distance 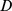, 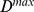, the statistical significance of this cut (at a desired significance level 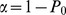) is verified by comparing 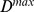 with the result that would be obtained by chance, given by the empirical curve 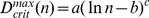, and 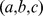  =  (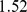, 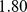, 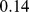) for 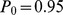, with 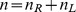. The signal is then split into two segments if 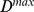 exceeds its critical value for the selected significance level, 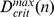. The procedure is then applied to each one of the segments, starting from the full series 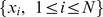, where 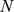 is the total number of data points, until no segmentable patches are left. (See Refs. [16], [17] for further details). We performed the KS-segmentation with 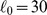, the minimal segment length, and 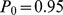. The choice of 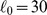 is based on its correspondence to the higher edge frequency of the very low frequency band of the heart rate with 0.03 Hz [18].

**Figure 1:**
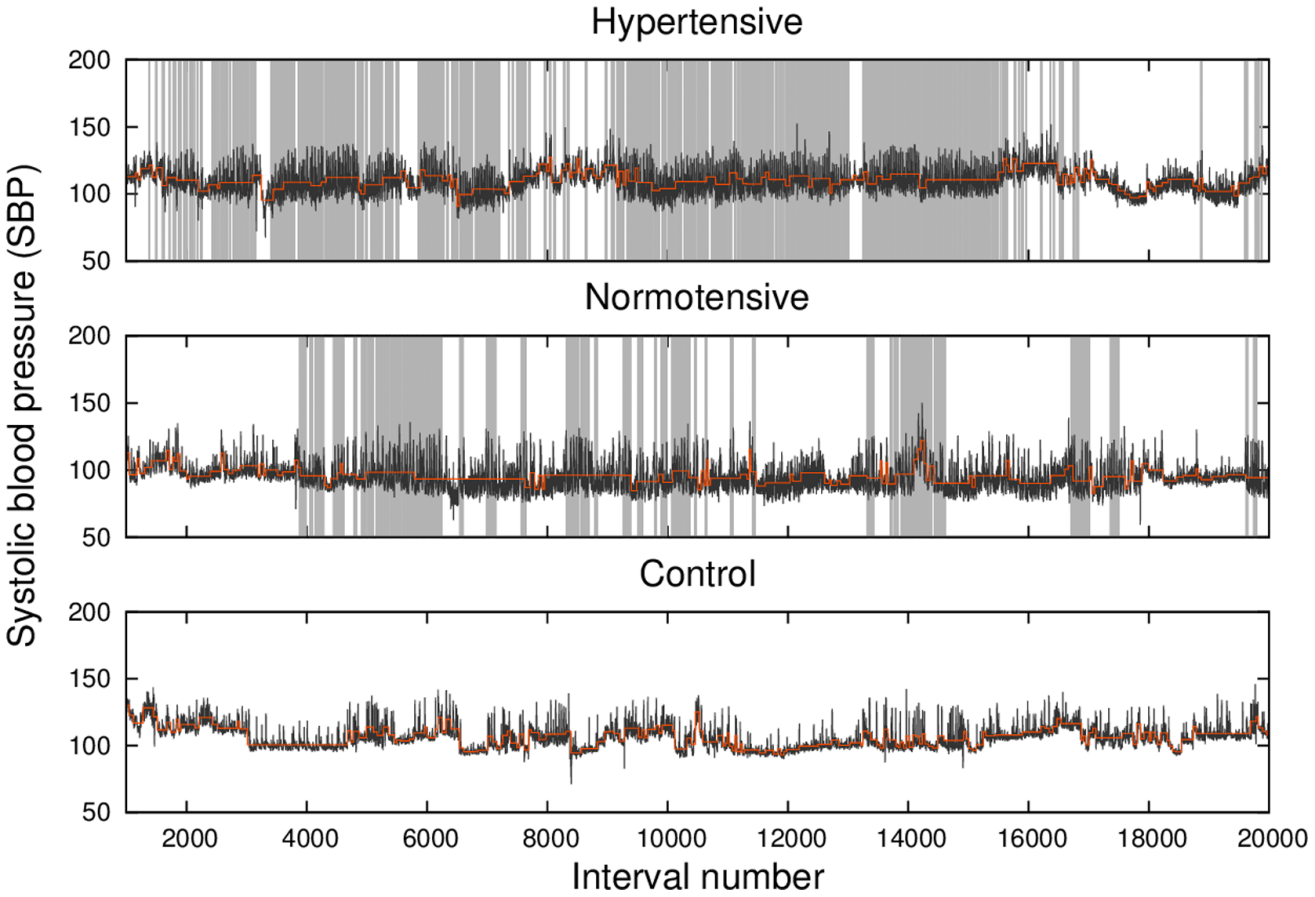
Systolic blood pressure (SBP) time series (black line) and its local mean values from segmentation (light orange line) for typical hypertensive (upper panel), normotensive (middle panel) and control (lower panel) subjects. Apnea events, detected via a polysomnography examination, are represented by the light gray vertical lines.

Fig. 1 shows the time series of SBP (black lines) for typical members of hypertensive (upper panel), normotensive (middle panel), and control (lower panel) groups, with the first and second patients suffering from sleep apnea-hypopnea. The local mean value of the signal in each data segment produced by the KS-segmentation procedure, 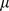, is also represented (light orange lines) in order to enable the reader to identify the stationary patches. For comparison, the sleep apnea events detected via polysomnography are also represented (light gray vertical lines).

## Results

**Figure 2:**
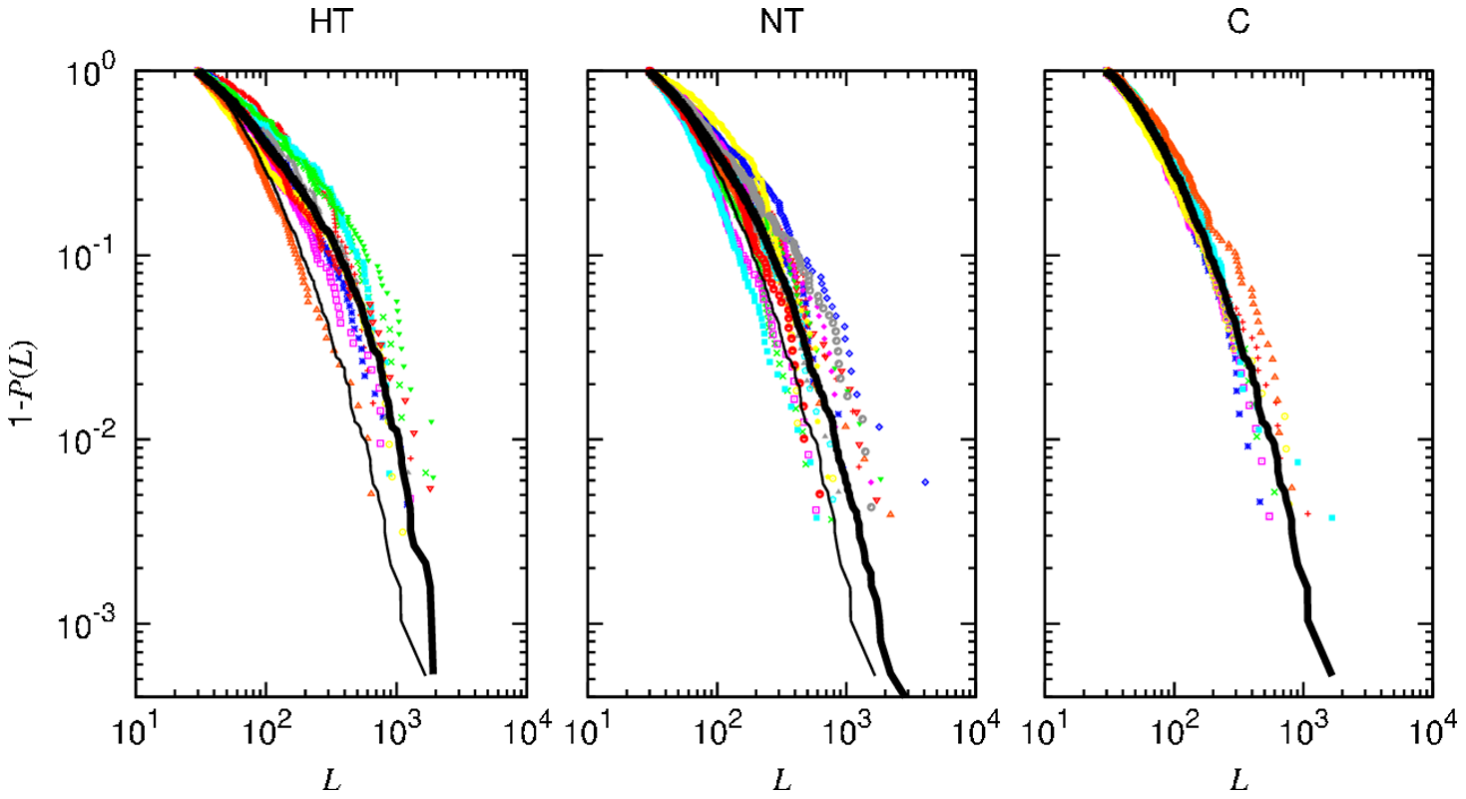
Complementary cumulative distribution of segment lengths for each individual (color) and accumulated data of all subjects in the same group (black solid line), for the hypertensive (HT), normotensive (NT) and control (C) groups. Drawn for comparison, the thin line in the first two panels (HT and NT) reproduces the accumulated data curve for the control group (C).

The statistics of segment lengths and of the statistical moments of the signal within each stationary segment provide a segmentation portrait of a given time series. We show in Fig. 2 the complementary cumulative distributions of segment lengths 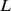, 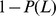, for SBP time series. The panels in the figure correspond to the hypertensive (HT), normotensive (NT) and control (C) groups. For each group, we show the length distribution taking into account the segments from each time series only (color symbols) and from all the time series of the same group (solid black lines).

**Figure 3:**
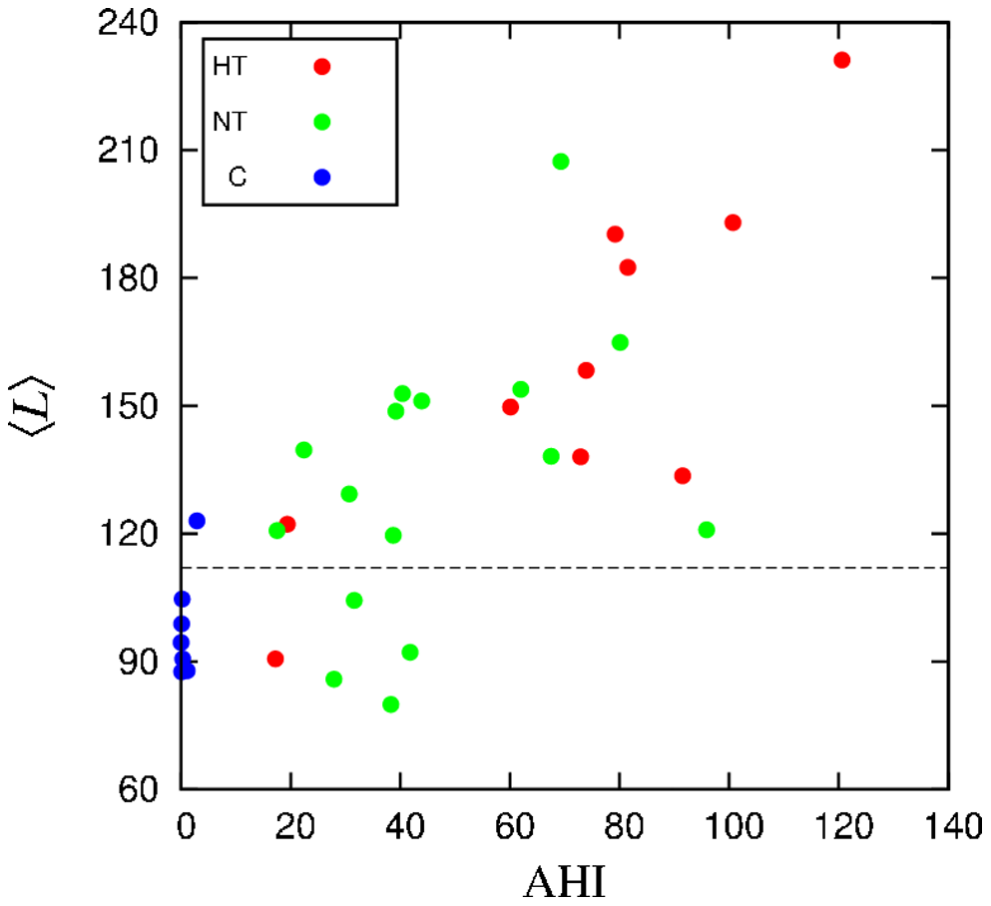
Mean length of the segments 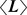 versus AHI for each subject. The dashed horizontal line represents the threshold value obtained by a ROC analysis.

Drawn for comparison, the thin line in panels HT and NT reproduces the curve in panel C corresponding to the control group accumulated data. A significant difference exists between the distribution of each apneic group and that of the control group (thick and thin black lines, respectively, in panels HT and NT), both cases yielding 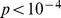 in the two-sample KS test. Moreover, we considered the set of values of 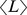 for each group (one value for each individual) and carried out the two-sample KS test to compare each apneic group with the control one. The *p*-values obtained (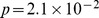 and 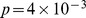, for the NT vs C and HT vs C, respectively) allow to reject the null hypothesis of equal distributions at a confidence level above 95% in both cases. This result points to 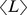 as a candidate to allow separation of apneic and control groups. In order to inspect a possible correspondence between the typical duration of stationary segments and degree of apnea, we plot in Fig. 3 the mean segment length 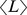 vs AHI, for each patient. As a matter of fact, a positive correlation between 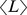 and AHI comes out (quantitatively, the Pearson coefficient is 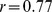). Moreover, one can set a threshold allowing to separate most apneic individuals. The threshold was chosen by minimizing the fractions of false negative and false positive results by means of ROC (receive operating characteristic) analysis [19]. This threshold allows to identify 69% of apneic subjects. Even so, we will investigate other quantities that might provide a similar or better separation.

**Figure 4:**
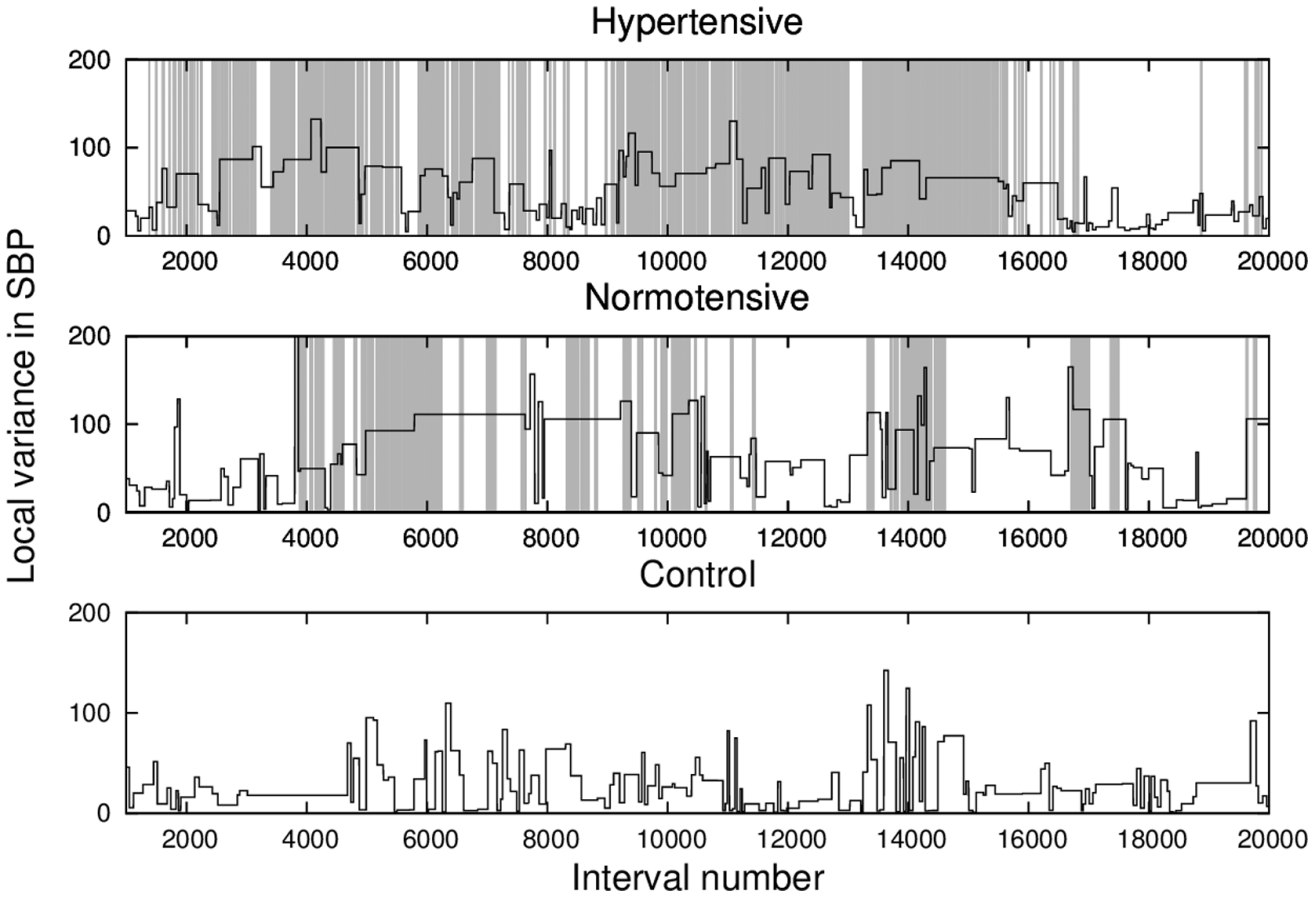
Local variance (black lines) provided by the segmentation of SBP and the standard apnea detection represented by the light gray lines, for the same examples of **Fig. 1.**

Let us investigate the statistics of the SBP signal in stationary segments. Since there is a tendency that apneic patients typically have larger variance on average, then the mean variance is a natural candidate for separation. In Fig. 4, we depict the local variance 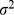 (variance of each patch) for one individual of each group. Notice that apnea epochs have a strong influence on the variability of the SBP signal, with higher dispersion (variance) in the gray areas, when compared to the scored apnea, both for the hypertensive and normotensive cases.

**Figure 5:**
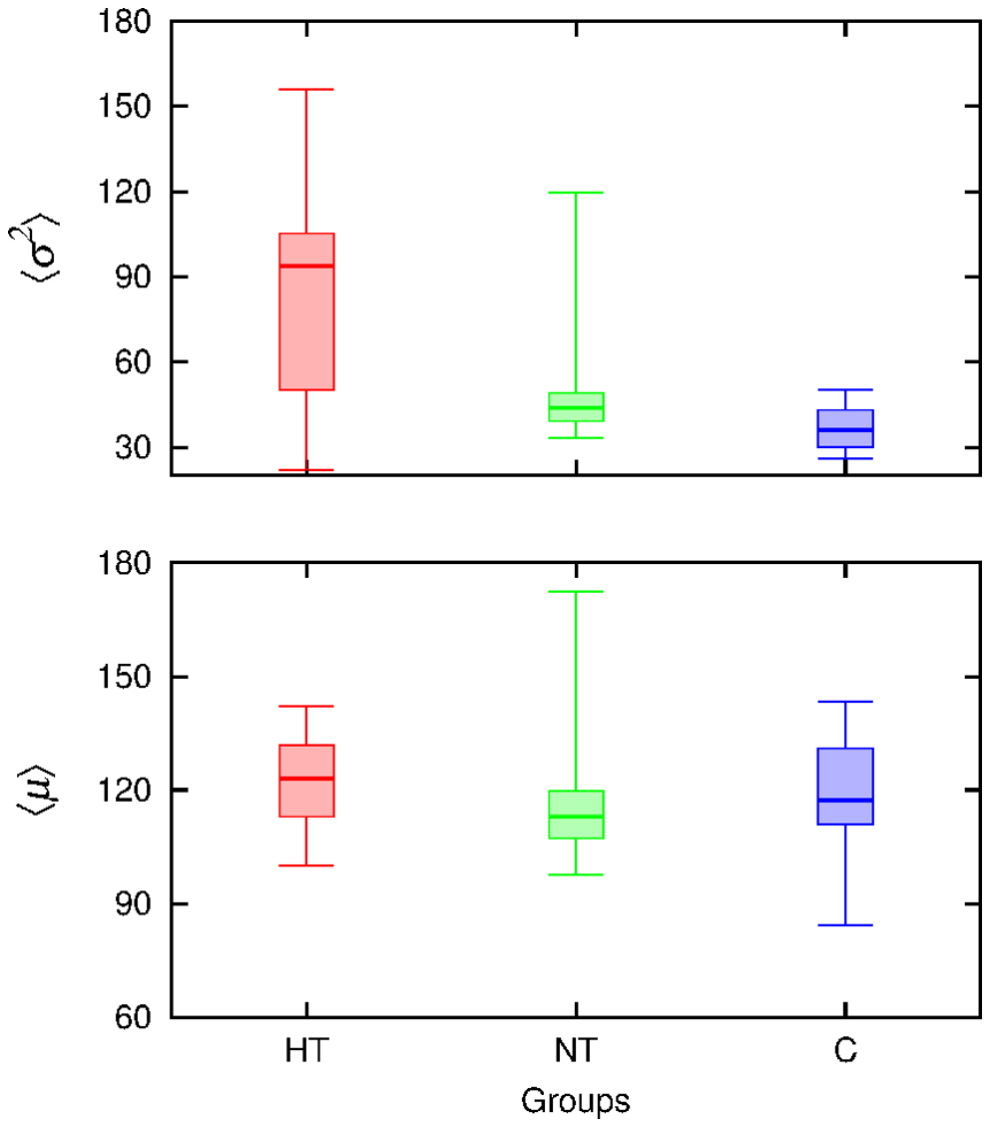
Quartiles of the distribution of the average variance 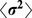 and of the average mean, 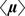, within each group. The horizontal lines limit the quartiles, the thicker one indicates the median.

**Figure 6:**
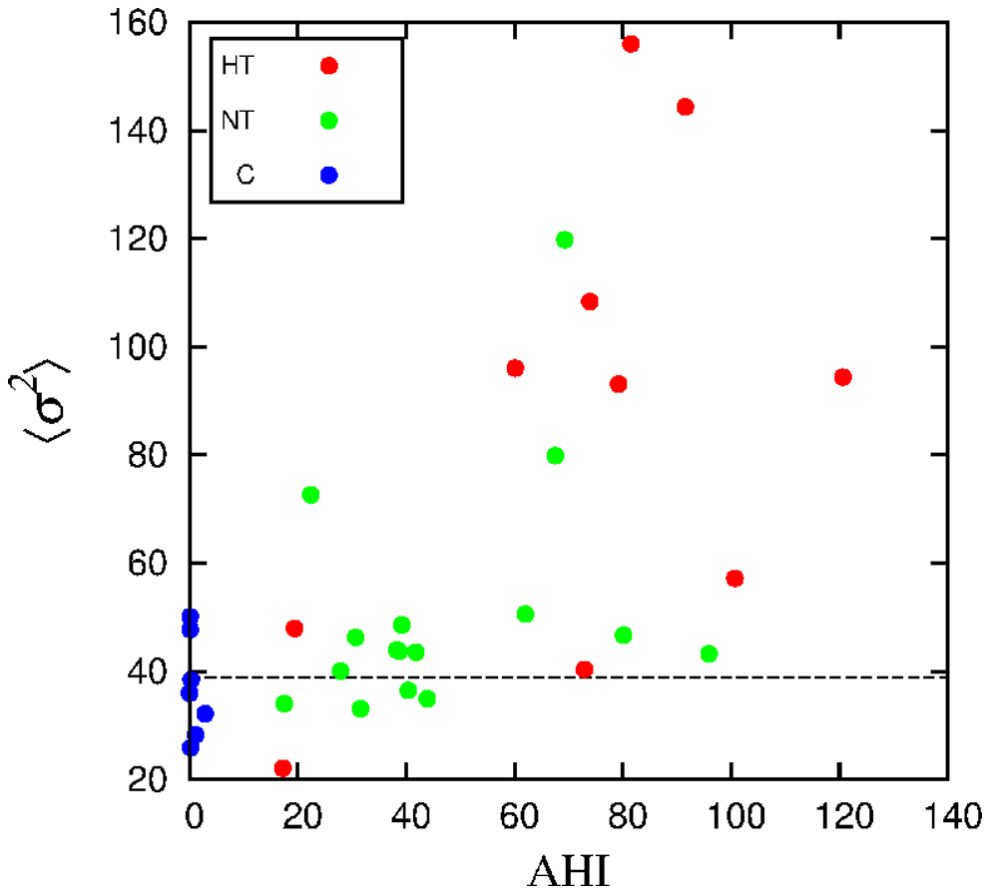
Mean variance of the segments 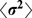 versus AHI for each subject. The dashed horizontal line represents the ROC threshold.

Like in the case of 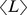, we performed the two-sample KS test to compare the sets of values of the average local variance 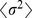 for each group. The *p*-values obtained (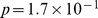 and 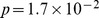, for NT vs C and HT vs C, respectively) do not allow to reject the null hypothesis in the first case. We further computed the quartiles (the three points that define the four equiprobable intervals) of 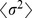, shown in Fig. 5. Although the control group presents a lower variability, there is too much overlap for a clear group separation. This is reinforced by the observation of a relatively weak correlation between the mean variance and AHI, as exhibited in Fig. 6 (with 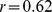). However, the ROC threshold allows to separate 69% of apneic subjects.

The average local mean 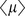 is less efficient (with stronger overlap) than the average variance for separability purposes (see Fig. 5). In fact, by means of the two-sample KS-test, we compared the sets of values of 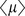 of each apneic group vs the control group, yielding 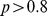 in both cases. Then, the null hypothesis of equal distributions can not be rejected at reasonable confidence levels. Similarly, when considering higher order moments, no significant differences amongst groups were detected.

The same analysis for the beat-to-beat series, BBI-BP and BBI-EKG, displayed weak correlations between the average length 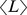 and AHI, with Pearson coefficient 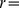 0.11 and 0.05, respectively. Also a weak correlation between the mean variance 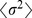 and AHI was observed, with 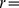 0.05 and 0.03, respectively. This is why we concentrated on blood pressure, as a potential candidate for sleep apnea diagnosis.

**Figure 7:**
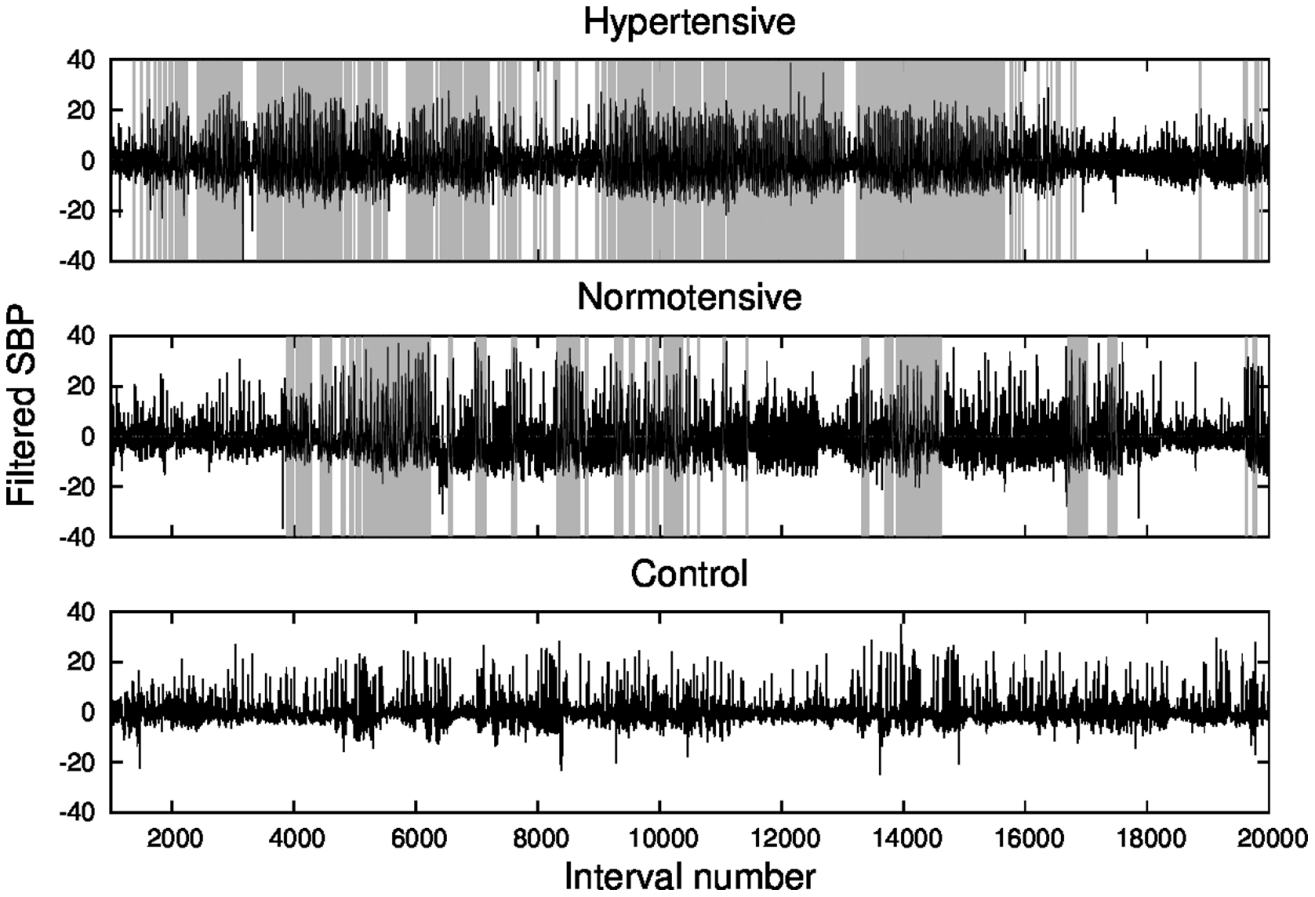
Systolic blood pressure time series, filtered by subtracting the local mean 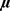 (black lines), and standard apnea detection (light gray vertical lines).

The next step is to look at time series autocorrelations. In order to obtain a signal that can be analyzed through standard spectral methods, we subtracted the local mean 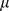 (the mean value of each data segment) from the values of the time series in the corresponding patch, yielding a filtered signal, shown with black lines in Fig. 7. The removal of the local average does not guarantee stationarity, because variance (e.g., Fig. 7) and higher-order moments may still change. However, it furnishes a detrended signal more stationary than the original one.

**Figure 8:**
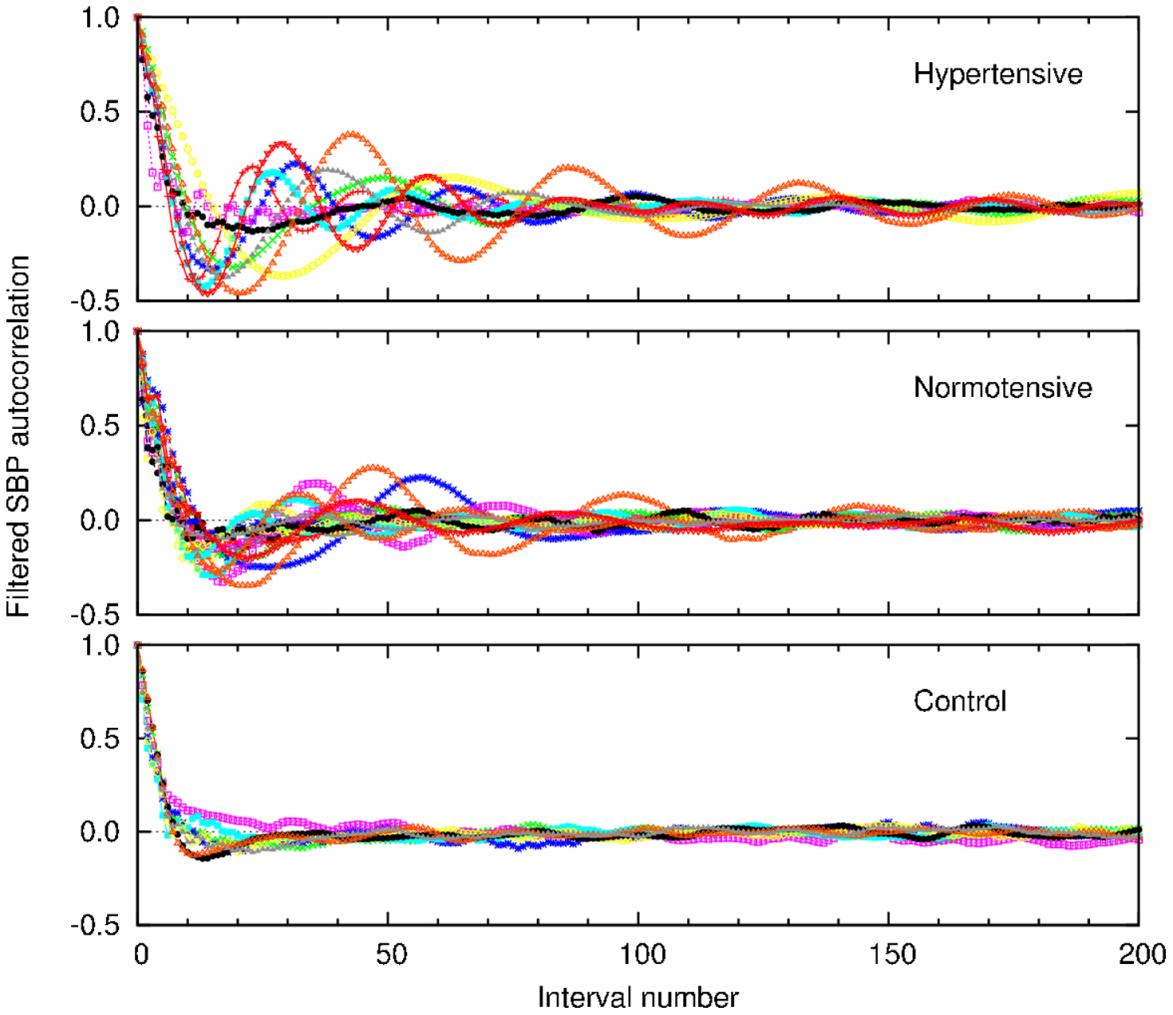
Autocorrelation of the filtered SBP time series, computed for all the individuals of each group.

Then we look at the behavior of the autocorrelation function of the filtered signal for each individual of each group, shown in Fig. 8. The autocorrelation function displays a remarkable behavior with oscillatory patterns, which are more pronounced in patients with sleep apnea, while it rapidly vanishes for the control group.

**Figure 9:**
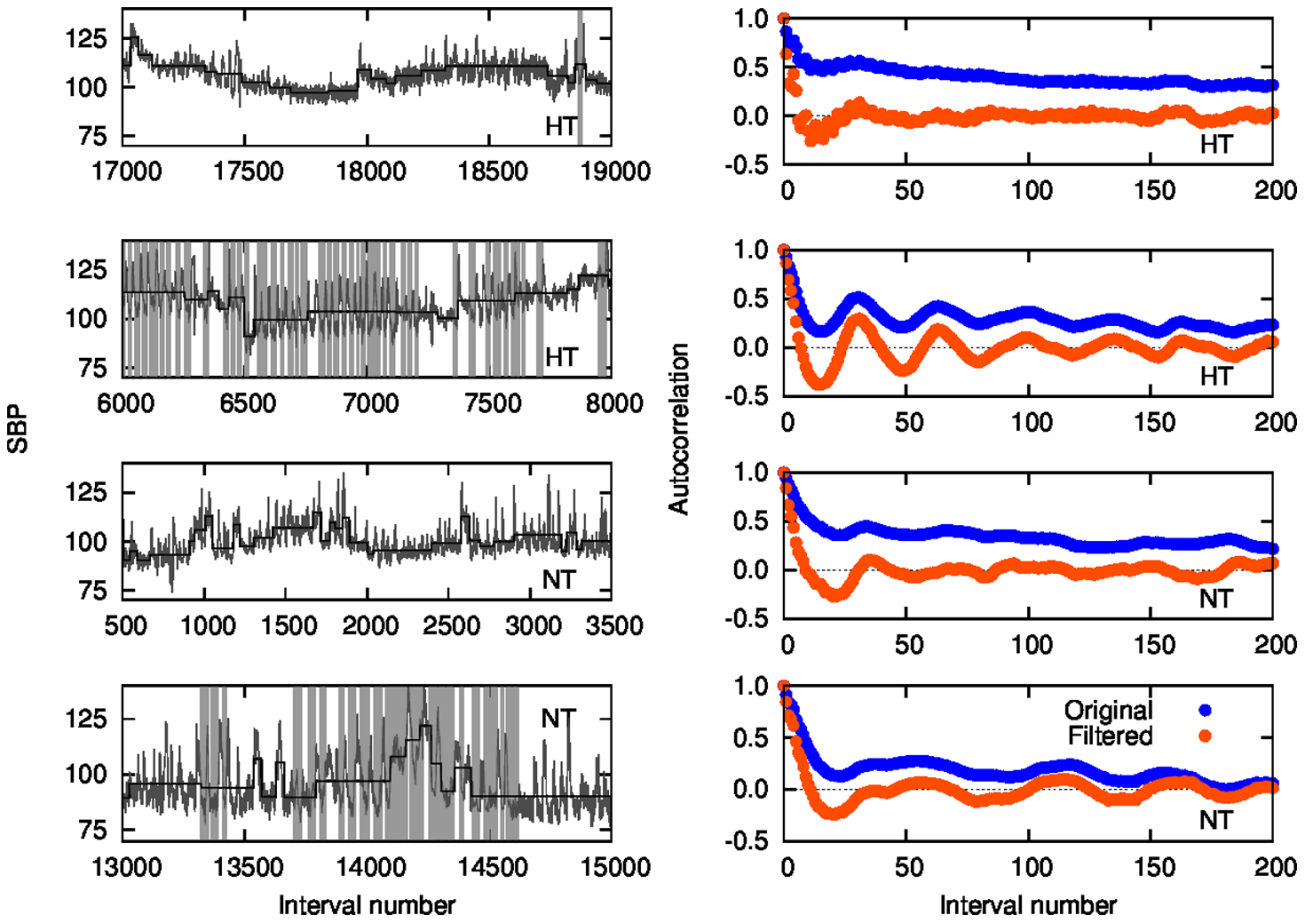
Left panels: Patches during non-apnea and apnea epochs (recognizable by the absence/presence of gray vertical lines), for hypertensive and normotensive subjects. Right panels: the corresponding autocorrelation function for the original and filtered (local mean 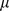 subtracted) signals.

According to the apnea score, the hypertensive subjects are in apnea on average during 28% of the records, while the normotensive ones are in apnea for 17%, on average. Thus, if one considers the whole times series, the effects of sleep apnea may be attenuated, particularly in the case of correlations. Then, we look at the autocorrelation function for two fragments of the SBP time series: 2000 points during sleep apnea epochs and 2000 points in a non-apnea epoch, in order to compare the effects of apnea in the same patient. Fig. 9 shows the autocorrelation function for the original and filtered (local mean subtracted) signals. Clearly, oscillations are related to apnea epochs. From the autocorrelation analysis, we conclude that the smaller amplitude of the oscillations observed in normotensive apneic subjects is not due to normal pressure but to a lower fraction of apnea epochs, then pointing apnea as the source of the oscillations regardless of the blood pressure condition.

**Figure 10:**
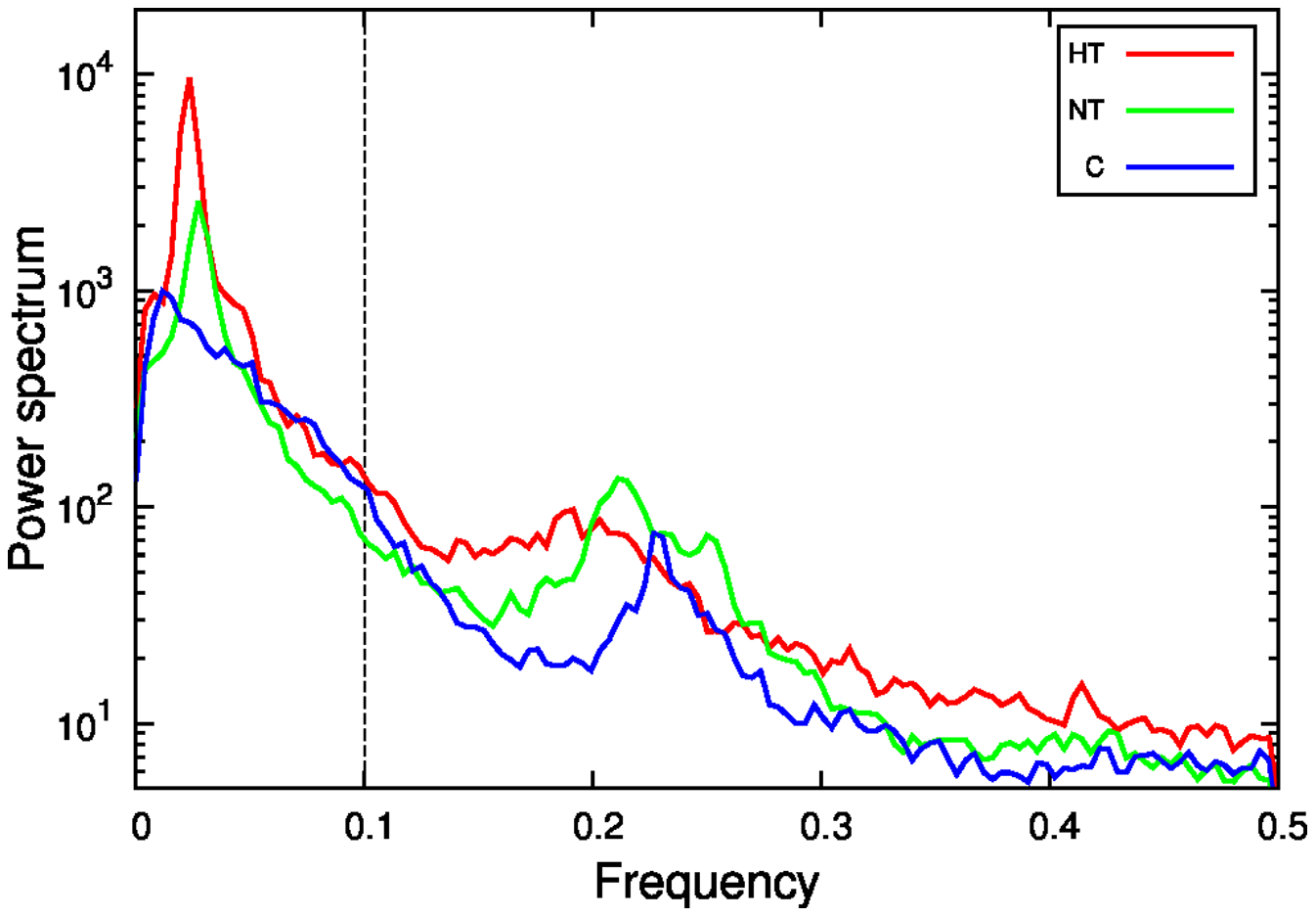
Power spectrum of the filtered SBP signal, for a typical individual of each group. Frequency corresponds to inverse interval number.

**Figure 11:**
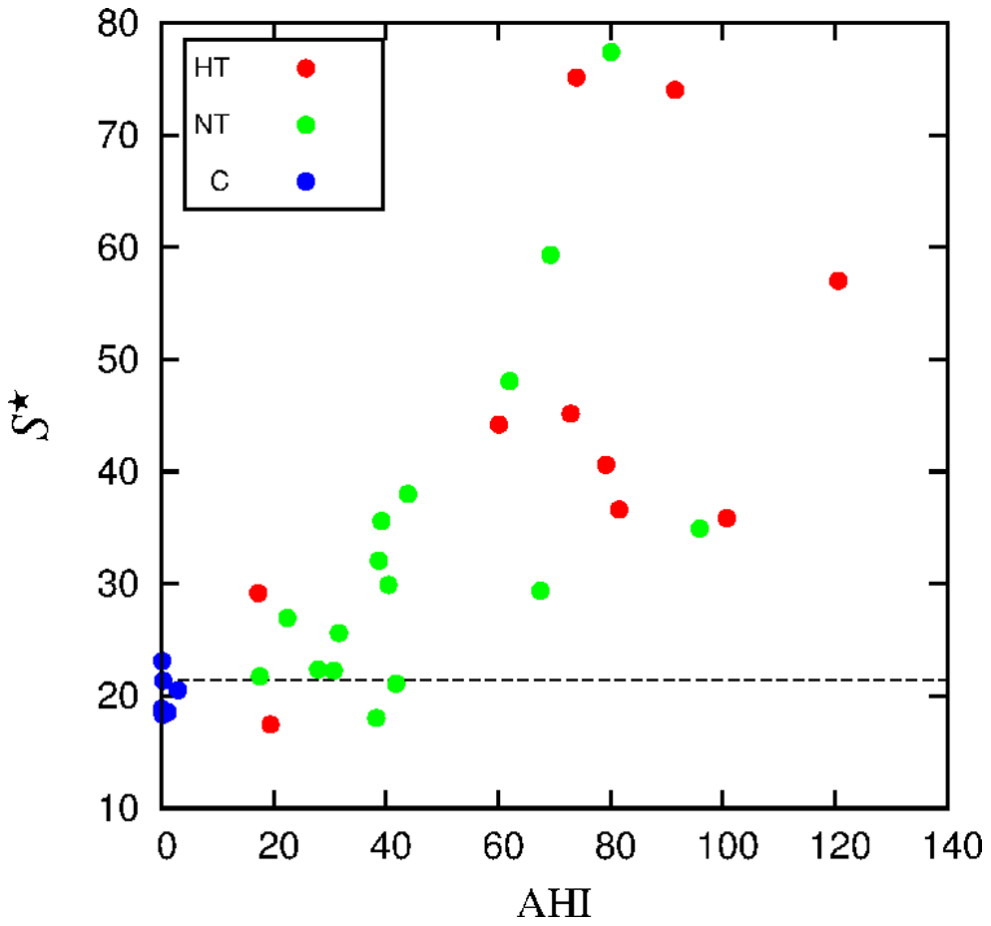
Normalized maximum 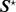 of the power spectrum versus AHI, for each individual. The dashed horizontal line represents the ROC threshold.

In order to properly characterize the oscillations, we proceed to obtain the spectral density of the filtered signal. To compute the spectral density we used Octave software, with pwelch function [20]. As illustrated in Fig. 10, the power spectrum manifests a main peak localized at a frequency about 0.02 in units of inverse interval number. In fact, the peak is typically more pronounced in apneic subjects. Then, according to the discussion in the precedent paragraph concerning the lower amplitude of the oscillations in normotensive subjects, we define the relative amplitude 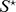, which is the maximum value of the amplitude normalized by the integral of the spectrum in the interval 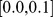. In Fig. 11, we represent 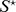 against the apnea index, exhibiting the correlation between both quantities (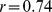).

Spectral analysis of interbeat interval increments has been previously carried out [21], [22]. In that case, the relative percentage of the very low frequency-component was taken as quantifier. However a ROC curve analysis presented a worse classification than in our case.

**Figure 12:**
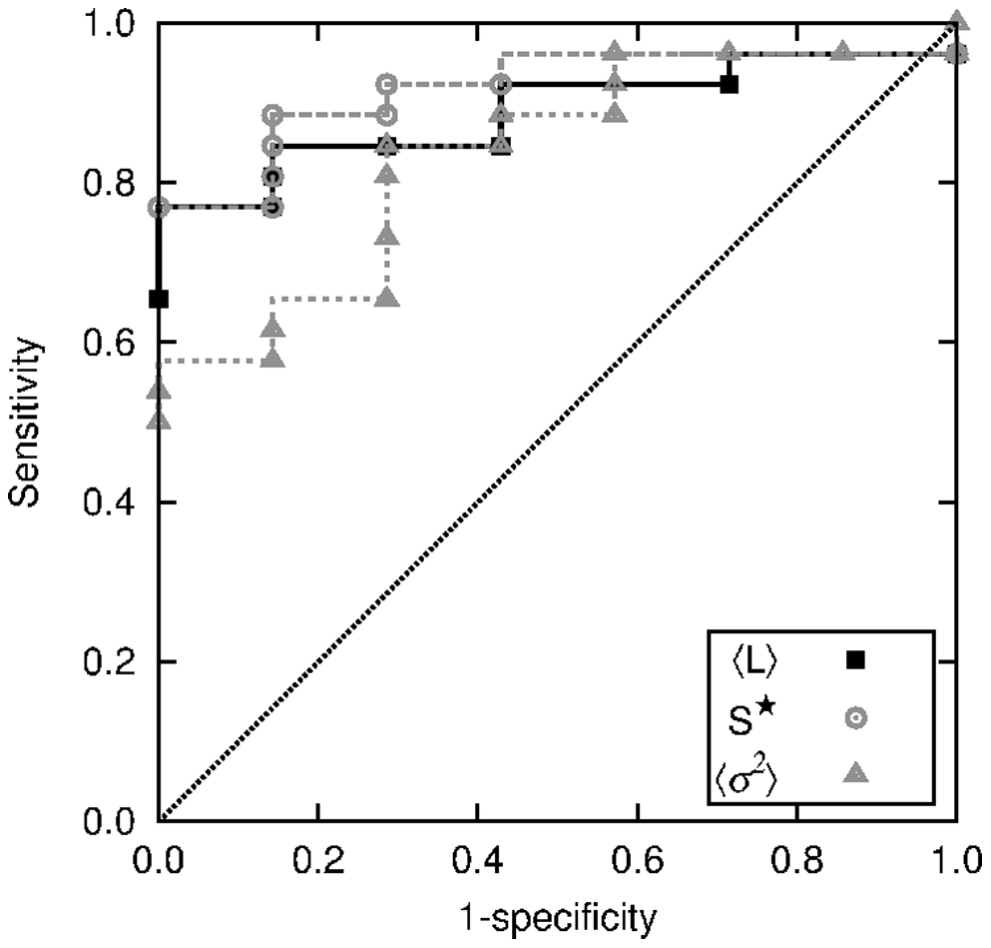
ROC curves for 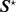, 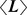 and 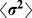, obtained to identify patients with apnea. Threshold values (21.4, 112 and 39, respectively) shown in previous figures were obtained from the optimal classification corresponding to the lowest distance to the upper left corner.

The ROC curves for the three quantities here considered as potential quantifiers, namely 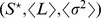, are displayed in Fig. 12. The respective thresholds were extracted from those curves. The accuracy (the sum of true positive and true negative subjects divided by the total sample size) of the optimal thresholds 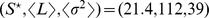 was 88, 82 and 79%, respectively.

**Figure 13:**
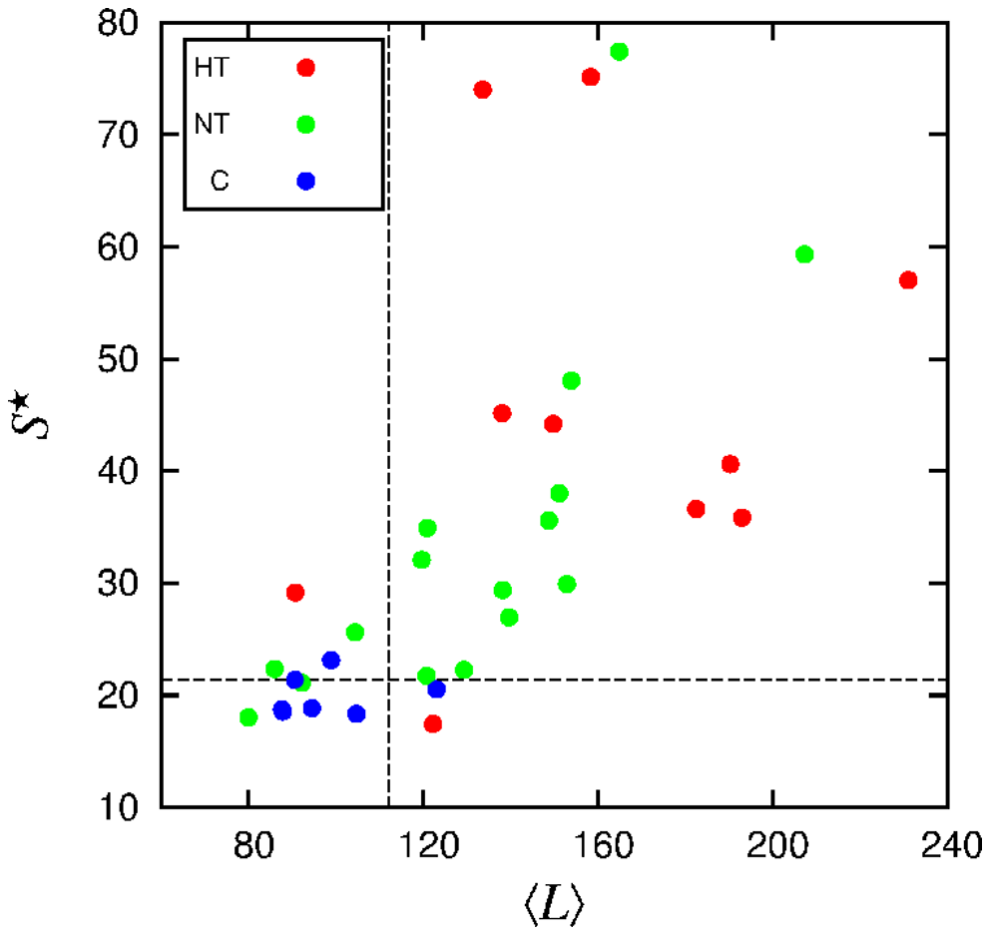
Normalized maximum of the spectral density 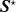 vs mean segment length 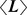, for each individual. Dashed lines represent threshold values.

The two quantities displaying higher Pearson coefficient, 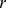, with respect to the apnea index AHI are 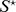 and 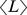. Then we combine them to obtain the diagram shown in Fig. 13. We observe a neat separation of non-apneic subjects in the low 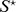 and low 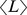 region.

## Discussion

Given the relevance of the diagnosis of sleep disturbances, particularly of sleep apnea, we look for an alternative procedure for detecting sleep apnea from simpler recordings than those composing a polysomnography, that yet must be performed in specialized sleep centers. For that purpose, we chose cardiovascular data as good candidates to furnish diagnosis elements. However, the nonstationary behavior of cardiovascular time series hampers the use of standard techniques that require stationarity. To deal with the nonstationarities, we applied a recently developed segmentation procedure [16], which allows the identification of patches of stationary behavior. Hence, for each patch, local quantities such as the statistical moments can be obtained. Let us also remark that a similar approach could be applied to other physiological signals, where nonstationarity is common. We analyzed the distribution of the length of stationary segments, as well as the SBP average mean and variance in such segments. Our results on the complementary cumulative distribution of segment lengths (Fig. 2) show that in the control group long patches are less probable, which reflects a more active blood pressure regulation in a healthy person. We found that the mean segment length 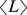 is correlated to AHI. Although weaker, there is also a correlation of the average local variance 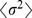 with AHI, where apnea epochs have a strong influence on the variability of the SBP signal. By subtracting the local mean 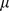, one obtains a modified signal which is more suitable than the original one, for instance, for spectral analysis. The autocorrelation function of the filtered signal displays a noticeable behavior, with oscillatory patterns, which are more pronounced in subjects with sleep apnea, while rapidly vanish for the control group.

Through segmentation, we detected features of the SBP signal that are correlated with apnea events. The main features are the relative intensity 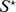 of the dominant oscillations in the autocorrelation function, the mean segment length 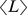, and, in a less extent, the average variance 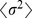. These quantities furnish criteria for the detection of apnea from SBP time series. According to the ROC curves, each of these quantities already shows a better performance than previous proposals based on heart rate increments where the sensitivity was lower than 75% [21], [22]. Particularly, the combination of two quantities, such as the mean segment length and the intensity of the oscillations, as shown in Fig. 13, allow a better evaluation.

As we have shown in a previous work [17], congestive heart failure is associated with a decreased variance of the segments in the time series of RR-intervals. In the present study, patients with such kind of diseases were not included, meaning that all the 26 patients considered did not suffer from heart rhythm disturbances. However, in future work we intend to evaluate how the presence of this cardiovascular disorder would affect the sleep-apnea oscillatory patterns and thresholds.

Our data comprise obstructive, central, and mixed sleep apnea events, with a majority of the events of obstructive type, except four subjects, that present a predominance of central type events. Our method was not designed to identify different types of apnea events. Even so, we carefully examined whether the type of apnea would be responsible for a particular regroupment in the diagrams as a function of AHI, but the four subjects are randomly distributed in all the diagrams. Then, another future direction would be to investigate the possibility of distinguishing different types of sleep apnea, such as central and obstructive types, by analyzing cardiovascular data.

Another relevant perspective would be to improve the thresholds by using larger samples and different sets of patients. However, we believe that the present results already give a valuable contribution in revealing measurable quantities that are correlated with AHI and in furnishing a novel criterion for diagnosis. Although improvements are welcome, our results furnish threshold values that can be used for diagnosis purposes. Once given a SBP time series, it can be handled by means of a code for segmentation, providing a diagnosis and its accuracy level.

Taking into account that a polysomnography is a labor intensive procedure, that involves many signals obtained under controlled settings in a sleep center, additionally requiring a visual analysis of all recorded signals by trained sleep experts, then an automated procedure as the one proposed here would be helpful.

## References

[1] Penzel T, Wessel N, Riedl M, Kantelhardt JW, Rostig S, et al (2007) Cardiovascular and respiratory dynamics during normal and pathological sleep. Chaos 17: 015116. 17411273 10.1063/1.2711282

[2] Stickgold R (2005) Sleep-dependent memory consolidation. Nature 437: 1272. 16251952 10.1038/nature04286

[3] Smith C (1995) Sleep states and memory processes. Behav Brain Res 69: 137. 7546305 10.1016/0166-4328(95)00024-n

[4] Penzel T, McNames J, de Chazal P, Raymond B, Murray A, et al (2002) Systematic comparison of different algorithms for apnoea detection based on electrocardiogram recordings. MEDICAL & BIOLOGICAL ENGINEERING & COMPUTING 40: 402–407. 12227626 10.1007/BF02345072

[5] Helland VCF, Gapelyuk A, Suhrbier A, Riedl M, Penzel T, et al (2010) Investigation of an automatic sleep stage classification by means of multiscorer hypnogram. Methods Inf Med 49: 467. 20644896 10.3414/ME09-02-0052

[6] Caples SM, Gami AS, Somers VK (2005) Obstructive sleep apnea. Annals of Internal Medicine 142: 187–197. 15684207 10.7326/0003-4819-142-3-200502010-00010

[7] Al-Angari HM, Sahakian AV (2007) Use of sample entropy approach to study heart rate variability in obstructive sleep apnea syndrom. IEEE TRANSACTIONS ON BIOMEDICAL ENGINEERING 54: 1900. 17926691 10.1109/TBME.2006.889772

[8] Penzel T, Kantelhardt JW, Grote L, Peter JH, Bunde A (2003) Comparison of detrended fluctuation analysis and spectral analysis for heart rate variability in sleep and sleep apnea. IEEE TRANSACTIONS ON BIOMEDICAL ENGINEERING 50: 1143. 14560767 10.1109/TBME.2003.817636

[9] Mendez M, Ruini D, Villantieri O, Matteucci M, Penzel T, et al. (2007) Detection of sleep apnea from surface ecg based on features extracted by an autoregressive model. In: Engineering in Medicine and Biology Society, 2007. EMBS 2007. 29th Annual International Conference of the IEEE. pp. 6105–6108. 10.1109/IEMBS.2007.4353742 18003408

[10] Canisius S, Ploch T, Gross V, Jerrentrup A, Penzel T, et al (2008) Detection of sleep disordered breathing by automated ecg analysis. Conf Proc IEEE Eng Med Biol Soc 2008: 2602–5. 19163236 10.1109/IEMBS.2008.4649733

[11] Wessel N, Malberg H, Bauernschmitt R, Kurths J (2007) Nonlinear methods of cardiovascular physics and their clinical applicability. International Journal of Bifurcation and Chaos 17: 3325.

[12] Suhrbier A, Heringer R, Walther T, Malberg H, Wessel N (2006) Comparison of three methods for beat-to-beat-interval extraction from continuous blood pressure and electrocardiogram with respect to heart rate variability analysis. Biomed Tech 51: 70. 10.1515/BMT.2006.013 16915768

[13] Gapelyuk A, Riedl M, Suhrbier A, Krmer J, Bretthauer G, et al (2011) Cardiovascular regulation in different sleep stages in the obstructive sleep apnea syndrome. Biomed Tech (Berl) 56: 207. 21823997 10.1515/BMT.2011.018

[14] Suhrbier A, Riedl M, Malberg H, Penzel T, Bretthauer G, et al (2010) Cardiovascular regulation during sleep quantified by symbolic coupling traces. Chaos 20: 045124. 21198136 10.1063/1.3518688

[15] Penzel T, Riedl M, Gapelyuk A, Suhrbier A, Bretthauer G, et al (2012) Effect of cpap therapy on daytime cardiovascular regulations in patients with obstructive sleep apnea. Comput Biol Med 42: 328. 21939968 10.1016/j.compbiomed.2011.09.001

[16] Camargo S, Queirós SMD, Anteneodo C (2011) Nonparametric segmentation of nonstationary time series. Phys Rev E 84: 046702. 10.1103/PhysRevE.84.046702 22181302

[17] Camargo S, Riedl M, Anteneodo C, Wessel N, Kurths J (2013) Diminished heart beat nonstationarities in congestive heart failure. Frontiers in Physiology 7: 107. 10.3389/fphys.2013.00107 PMC3654225 23720631

[18] Malik M, Bigger JT, adn Robert E Kleiger AJC, Malliani A, Moss AJ, et al (1996) Guidelines - heart rate variability. European Heart Journal 17: 354.8737210

[19] Metz CE (1978) Basic principles of roc analysis. Seminars in Nuclear Medicine VIII: 283. 10.1016/s0001-2998(78)80014-2 112681

[20] Octave-Forge Documentation website. Available: http://octave.sourceforge.net/signal/function/pwelch.html. Accessed 2014 August 22.

[21] Poupard L, Court-Fortune I, Pichot V, Chouchou F, Barthlmy JC, et al (2011) Use of high-frequency peak in spectral analysis of heart rate increment to improve screening of obstructive sleep apnoea. Sleep and Breathing 15: 837–843. 21104152 10.1007/s11325-010-0446-0

[22] Roche F, Sforza E, Duverney D, Borderies JR, Pichot V, et al (2004) Heart rate increment: an electrocardiological approach for the early detection of obstructive sleep apnoea/hypopnoea syndrome. Clin Sci 107: 105–110. 14992679 10.1042/CS20040036

